# Editorial: Measurable brain and cognitive reserve: The implication of neuroimaging biomarkers in the normal aging process

**DOI:** 10.3389/fnagi.2022.1020435

**Published:** 2022-09-13

**Authors:** Chu-Chung Huang, Ching-Po Lin, Kenji Toba, Duan Xu

**Affiliations:** ^1^Shanghai Key Laboratory of Brain Functional Genomics (Ministry of Education), Affiliated Mental Health Center (ECNU), School of Psychology and Cognitive Science, East China Normal University, Shanghai, China; ^2^Shanghai Changning Mental Health Center, Shanghai, China; ^3^Institute of Neuroscience, National Yang Ming Chiao Tung University, Taipei, Taiwan; ^4^Brain Research Center, National Yang Ming Chiao Tung University, Taipei, Taiwan; ^5^Center for Healthy Longevity and Aging Sciences, National Yang Ming Chiao Tung University, Taipei, Taiwan; ^6^Tokyo Metropolitan Institute of Gerontology, Tokyo, Japan; ^7^Department of Radiology and Biomedical Imaging, University of California, San Francisco, San Francisco, CA, United States

**Keywords:** cognitive reserve, brain reserve hypothesis, neuroimage, aging, lifespan

Cognitive aging is universal, but individual trajectories vary widely. Many older adults are capable of maintaining good physical, psychological, and social functioning until the end of life, called “successful aging” (Rowe and Kahn, 1997). It is believed that these individuals are more resistant to neuropathological challenges, and their cognition ages more slowly. By observing the resilient nature of these “successfully aging” brains, “reserve” concepts are constructed to explain such heterogeneity among individuals.

In the theory of brain reserve (BR), individuals with higher brain volumes or neural resources are better able to resist pathological damage and aging-related changes (Satz, 1993). Meanwhile, cognitive reserve (CR) suggests that pre-acquired cognitive skills, such as IQ, education, occupational attainment, exercise, and leisure activities, enrich the brain's networks to cope with brain atrophy or damage (Stern, 2013). Both theories seek to explain individual heterogeneity. With the advent of neuroimaging techniques, recent studies have made significant progress in measuring brain capacity to predict one's cognitive aging trajectory and health status. This helps fulfill the BR and CR theories at the practical level (Cabeza et al., 2018; Groot et al., 2018; Cole et al., 2019; Stern et al., 2019).

In this Research Topic, advanced neuroimaging techniques were used to investigate or estimate the effects of CR in the normal aging process and populations with neurodegenerative disorders.

Given that the concept of CR is related to the cognitive resiliency of an individual's brain during neural challenges. Lee et al. utilized the AD neuropathology—tau, amyloid—and cortical thickness using T1-MRI to predict cognitive function, the residuals of which is conceptualized as CR to associate with disease progression. They demonstrated that the effect of CR can be different according to the disease status, that higher CR was related to a mitigated decline in CU individuals, while it was associated with exacerbated cognitive decline in the AD spectrum. Using FDG-PET, Kato et al. investigated the effect of CR on amnestic MCI. Among MCI patients showing clinical AD patterns based on Silverman's classification, glucose hypometabolism was observed in the high-education group compared with the low-education group. Furthermore, cognitive decline was more rapid in the high-education group over 3 years. Although using education as a proxy of CR remains debatable, this study indicates that individuals with higher CR may instead suffer a greater cognitive decline in the face of pathological brain changes. Since brain atrophy is often accompanied by cognitive decline, Imabayashi et al. demonstrated brain volume atrophy composite scores in medial temporal regions were deemed as early AD markers in the evaluation of early volume changes in cognitively normal participants (CN). They found that CN with positive amyloid instead shows enlarged left medial temporal volume than those without amyloid deposition, which might be explained by an increase in brain volume as a mechanism to compensate for AD pathology in the preclinical stage to maintain cognitive performance. Using multimodal neuroimaging techniques, Kim et al. proposed the negative behavioral reserve (nBR) hypothesis (predicted negative symptoms using neuroimaging markers) to explain individual variability in behavioral problems in frontotemporal dementia. Their findings suggest that participants with higher nBR have lower negative symptoms and are associated with lesser atrophy in the frontotemporal cortex and greater white matter integrity. These findings imply that individuals with greater BR may have fewer clinical symptoms than those with the same neuropathological burden. Toh and Siow utilized a new diffusion MRI algorithm that analyzes the diffusivity along perivascular spaces (ALPS) to assess glymphatic activity (Taoka et al., 2017), which is related to the efficiency of waste clearance in the brain. The ALPS index showed lower values in ischemic stroke patients than normal controls, suggesting impaired glymphatic function. Additionally, the ALPS index can increase with time since stroke onset, suggesting glymphatic function recovery. This study provides evidence for assessing brain health using diffusion-based ALPS estimates.

The studies by Lee et al., Kato et al., Imabatyshi et al., and Toh and Siow provide neuroimaging evidence for individuals susceptible or resistant to neuropathology. Based on their findings, the effect of CR may vary depending on the disease spectrum stage. Future studies should consider the compensatory effect or the role of brain structural and functional changes longitudinally.

Hsieh and Yang investigated the association of diffusion parameter changes in brain white matter (WM) with cognition in 114 mid-aged adults within a 2-year interval. While some brain-cognition associations can be found in the cross-sectional dataset, only the association between WM integrity and processing speed could be replicated in both cross-sectional and longitudinal datasets. These findings support aging-related changes in processing speed over the 2-year interval and highlight the necessity of longitudinal design for studies of aging-related cognition. A longitudinal study by Pur et al. examined age-related changes in brain structural connectivity (SC) and functional connectivity (FC) within 2 years in older adults using diffusion-weighted imaging and resting-state functional MRI. They demonstrated that age-related cognitive declines could be supported by a distinct positive and negative contribution of SC and FC. The results suggest a tendency to preserve structural connections but decline functional ones during the cognitive aging process, which may imply the neural basis of cognitive reserve that protects against aging-related degeneration. Wei et al. compared the dynamic FC using resting-state fMRI between those with subjective cognitive decline (SCD) and a control group. The study demonstrated that changes in frontoparietal dynamic connectivity could occur early in the preclinical stage of SCD, which suggests the critical role of dynamic FC of FPN in balancing subjective and objective cognition.

Hsieh and Yang and Pur et al. focus on longitudinal changes from the brain network perspective. Wei et al. demonstrated the dynamic FC changes at the stage before objective cognition declines. These studies shed insight into cognitive reserve research using neuroimaging techniques, that balancing segregation and integration at the network level could be essential to brain health status instead of relying on traditional localizationism concepts.

Leisure activity (LA) engagement in later life has been associated with greater cognitive reserve; however, how it influences brain health remains unclear. Iizuka et al. classified 482 participants into four different subtypes of LA engagement according to how many types of LA they are currently engaged in. The results demonstrated that people who engaged in ≥3 types of LAs showed greater hippocampal volume and gray matter volume, which was more pronounced among males than females. Even though most domains of cognition decline with age, language is an exception that remains stable from adulthood to later life. In this regard, Yeh et al. examined whether different brain regions are in charge between young and older adults during idioms processing. They found that older adults showed higher accuracy for frequent idioms and equivalent accuracy for infrequent idioms than younger adults. In addition, older adults exhibited higher functional activations in the bilateral frontotemporal and medial frontoparietal regions. This study provides evidence for the alternative view that aging may not necessarily be solely accompanied by decline.

The cognitive reserve emphasizes the importance of acquired experiences throughout early lifetime. The studies by Iizuka et al. and Yeh et al. provide in-depth evidence in support of this hypothesis, utilizing neuroimaging measurements to explain individual differences in cognitive abilities.

The above contributions are but a few papers collected in this Research Topic addressing important issues about how reserve can be measured across the human lifespan using multimodal neuroimaging techniques. Future studies will endeavor to leverage the variety of neuroimaging methods to explain the heterogeneity of human behavior and cognition, and discover the underlying neural mechanism of the reserve. More importantly, interventional study designs should be considered to assess and clarify the neurobiological effects of mental, physical training or active lifestyles on cognitive reserve.

## Author contributions

C-CH drafted the manuscript. C-PL, KT, and DX reviewed it. All authors contributed to the article and approved the submitted version.

## Conflict of interest

The authors declare that the research was conducted in the absence of any commercial or financial relationships that could be construed as a potential conflict of interest.

## Publisher's note

All claims expressed in this article are solely those of the authors and do not necessarily represent those of their affiliated organizations, or those of the publisher, the editors and the reviewers. Any product that may be evaluated in this article, or claim that may be made by its manufacturer, is not guaranteed or endorsed by the publisher.
